# H2Bub1 loss is an early contributor to clear cell ovarian cancer progression

**DOI:** 10.1172/jci.insight.164995

**Published:** 2023-06-22

**Authors:** Adam J. Ferrari, Priyanka Rawat, Hannah S. Rendulich, Akshaya V. Annapragada, Yasuto Kinose, Xiaoming Zhang, Kyle Devins, Anna Budina, Robert B. Scharpf, Marilyn A. Mitchell, Janos L. Tanyi, Mark A. Morgan, Lauren E. Schwartz, T. Rinda Soong, Victor E. Velculescu, Ronny Drapkin

**Affiliations:** 1 Penn Ovarian Cancer Research Center, Department of Obstetrics and Gynecology;; 2 Graduate Program in Cell and Molecular Biology; and; 3 Department of Cancer Biology, University of Pennsylvania Perelman School of Medicine, Philadelphia, Pennsylvania, USA.; 4 Sidney Kimmel Comprehensive Cancer Center, Johns Hopkins University School of Medicine, Baltimore, Maryland, USA.; 5 Department of Pathology and Laboratory Medicine, Hospital of the University of Pennsylvania, Philadelphia, Pennsylvania, USA.; 6 Department of Pathology, University of Pittsburgh School of Medicine, Pittsburgh, Pennsylvania, USA.; 7 Basser Center for BRCA, Abramson Cancer Center, University of Pennsylvania Perelman School of Medicine, Philadelphia, Pennsylvania, USA.

**Keywords:** Cell Biology, Oncology, Cancer, Epigenetics, Obstetrics/gynecology

## Abstract

Epigenetic aberrations, including posttranslational modifications of core histones, are major contributors to cancer. Here, we define the status of histone H2B monoubiquitylation (H2Bub1) in clear cell ovarian carcinoma (CCOC), low-grade serous carcinoma, and endometrioid carcinomas. We report that clear cell carcinomas exhibited profound loss, with nearly all cases showing low or negative H2Bub1 expression. Moreover, we found that H2Bub1 loss occurred in endometriosis and atypical endometriosis, which are established precursors to CCOCs. To examine whether dysregulation of a specific E3 ligase contributes to the loss of H2Bub1, we explored expression of ring finger protein 40 (RNF40), ARID1A, and UBR7 in the same case cohort. Loss of RNF40 was significantly and profoundly correlated with loss of H2Bub1. Using genome-wide DNA methylation profiles of 230 patients with CCOC, we identified hypermethylation of *RNF40* in CCOC as a likely mechanism underlying the loss of H2Bub1. Finally, we demonstrated that H2Bub1 depletion promoted cell proliferation and clonogenicity in an endometriosis cell line. Collectively, our results indicate that H2Bub1 plays a tumor-suppressive role in CCOCs and that its loss contributes to disease progression.

## Graphical abstract

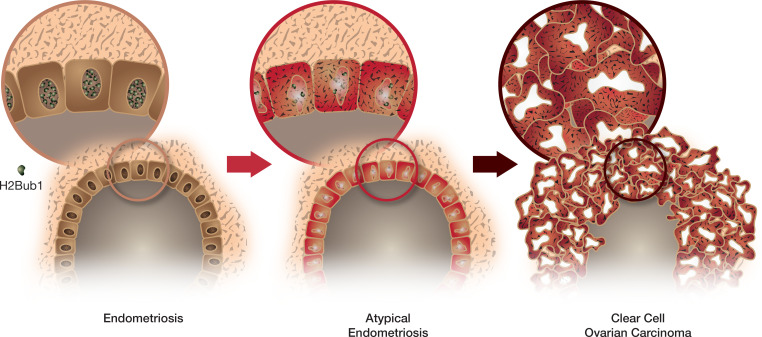

## Introduction

Epigenetic modifications are a major contributor to cancer development and progression. In human cancers, epigenetic changes can include aberrations in noncoding RNA expression, nucleosome remodeling, DNA methylation, and histone modifications (1–4). Importantly, histone modifications are manifold and can include monoubiquitylation of histones. Of particular interest is the monoubiquitylation of histone H2B (H2Bub1) (5–7). This posttranslational histone mark contributes to various cellular functions, including transcription elongation, DNA repair, stem cell differentiation, and histone crosstalk in normal tissues (8–13). However, in the context of diverse cancer types, H2Bub1 is often lost in malignant tissue compared with the benign counterpart. In some cases, its loss has been reported to contribute to more malignant phenotypes, and it is associated with worse outcomes (14–16).

H2Bub1 is regulated by E3 ligases that ubiquitinate the histone and deubiquitinase enzymes that remove the ubiquitin moiety (7, 10, 17). The ring finger protein 20 (RNF20)/RNF40 complex is the primary E3 ligase responsible for adding the single ubiquitin moiety to histone H2B at lysine 120 (7, 17). In addition to the RNF20/40 complex, AT-rich interaction domain 1A (ARID1A) and ubiquitin protein ligase E3 component n-recognin 7 (UBR7) have also been implicated in ubiquitinating H2B at lysine 120 (17, 18).

In the context of high-grade serous ovarian cancer (HGSOC), H2Bub1 is lost in approximately 75% of cases but is retained in benign fallopian tube epithelium, the primary site of origin for HGSOCs (19–22). Work from our laboratory has traced the loss of H2Bub1 to serous tubal intraepithelial carcinoma (STIC), the precursor to HGSOC (20), suggesting that it is an early event in tumor progression. Interestingly, it was observed that H2Bub1 depletion in fallopian tube secretory epithelial cells (FTSECs) increases their tumorigenic phenotypes, including migration and anchorage-independent growth (20). Mechanistically, H2Bub1 loss in FTSECs altered chromatin accessibility, leading to transcriptional upregulation of pro-inflammatory cytokines, including interleukin-6 and tumor necrosis factor-α (20). These results are consistent with animal studies in colorectal and lung cancers showing that H2Bub1 depletion results in a more inflamed tumor microenvironment and confirm that H2Bub1 correlates with worse outcomes in several cancer types (15, 23).

Although work from our group has focused on HGSOC, little is known about the role of H2Bub1 in other histologic subtypes of ovarian epithelial carcinomas. Here, we explore the status of H2Bub1 in clear cell ovarian carcinoma (CCOC), low-grade serous ovarian carcinoma (LGSOC), and endometrioid ovarian carcinomas. We show that H2Bub1 loss is common in all 3 histologic subtypes but is most profound in CCOC. Nearly all cases of CCOC exhibit low or no H2Bub1 expression. Moreover, we find that H2Bub1 is lost in precursors of CCOC, including endometriosis and atypical endometriosis (24–27), indicating that it is an early event. Mechanistically, we used genome-wide DNA methylation profiles to show that hypermethylation of *RNF40* is a major underlying cause of H2Bub1 loss in CCOC. Finally, we address the functional consequences of H2Bub1 loss on the oncogenic behavior of CCOC precursor cells.

## Results

### H2Bub1 is lost in ovarian cancer subtypes.

H2Bub1 loss is reported in approximately 75% of HGSOC tumor tissue and contributes to more malignant phenotypes in HGSOC precursor cells (19, 20). While this supports an important role for H2Bub1 loss in HGSOC pathogenesis, little is known about its contribution in other subtypes of ovarian cancer. In an effort to evaluate the status of H2Bub1 in other histotypes, we performed immunohistochemistry (IHC) analysis of 40 patient cases of CCOC, 44 cases of LGSOC, and 18 cases of endometrioid carcinoma for both H2B and H2Bub1. Cases were reviewed and scored semiquantitatively for nuclear staining intensity and percentage of positive cells. Subsequently, a composite score (CS) for each case was calculated and compared (Methods).

**Figure 1:**
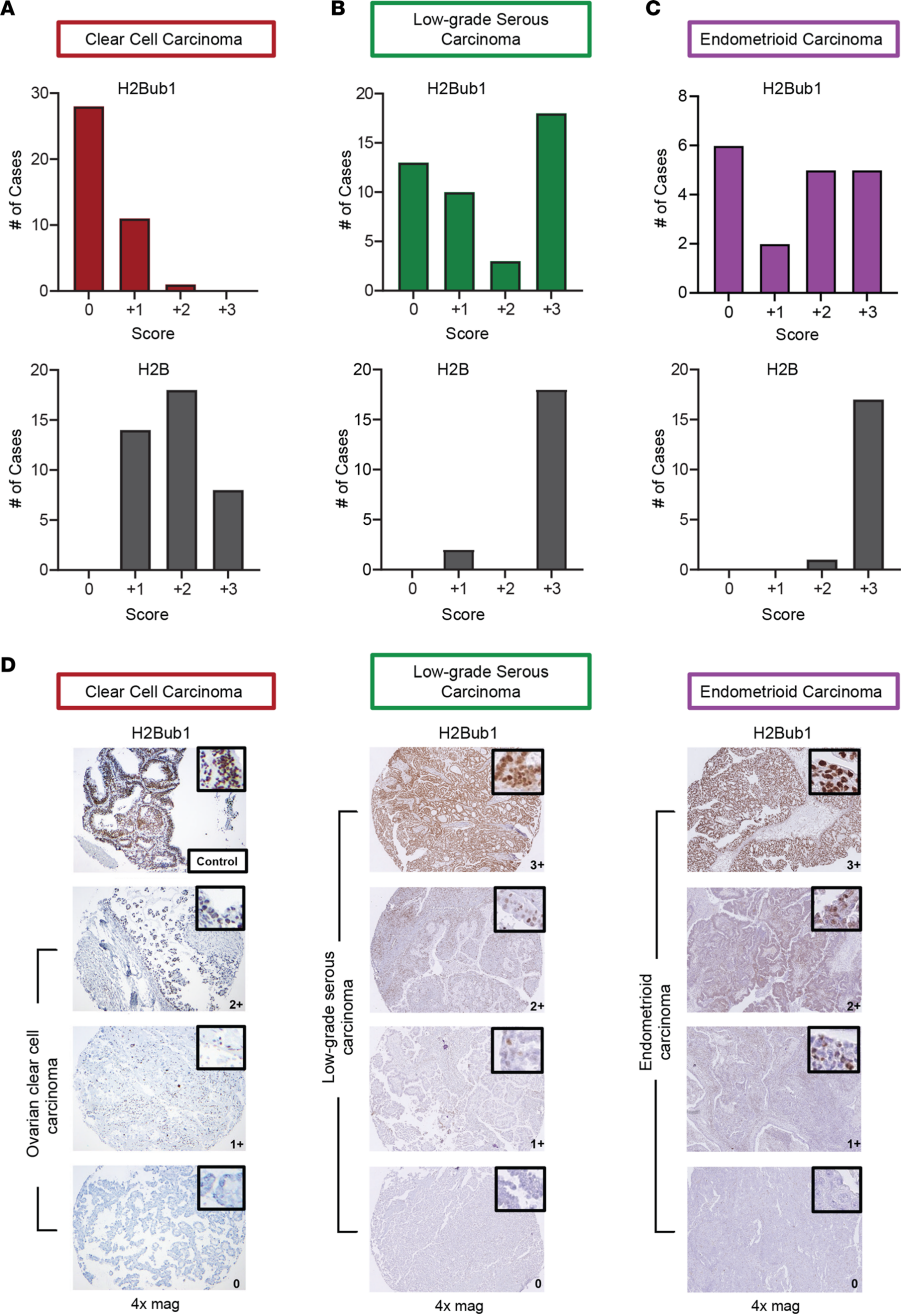
H2Bub1 expression in ovarian cancer subtypes. (**A**) Analysis of both H2Bub1 and the core histone control, H2B, in a tissue microarray (TMA) of 40 CCOC cases. (**B**) Analysis of both H2Bub1 and H2B in 44 LGSOC cases. (**C**) Analysis of both H2Bub1 and H2B in 18 cases of endometrioid carcinoma. (**D**) Representative images used in tissue scoring including a positive control (endometrioid carcinoma, top left).

The vast majority of CCOC showed weak to negative H2Bub1 immunostaining with 28/40 cases exhibiting a 0 CS and ultimately 39/40 (97.5%) cases showing weak to negative staining. In fact, only 1 case displayed +2 CS staining, and no cases represented a +3 CS. As expected, loss of the core histone H2B was not observed in any of the cases (Figure 1A and Supplemental Tables 1 and 2; supplemental material available online with this article; https://doi.org/10.1172/jci.insight.164995DS1). Out of 44 cases of LGSOC, 30% showed absent staining for H2Bub1 and 23/44 (52%) had negative to weak staining, while the core histone was present in a subset of samples evaluated (Figure 1B). Of the 18 endometrioid carcinomas, 6 showed complete H2Bub1 loss and 8/18 (44%) cases showed negative to weak staining (Figure 1C). IHC expression, where present, was restricted to the cell nucleus as expected (Figure 1D).

### H2Bub1 loss is associated with low levels of the E3 ligase RNF40 in CCOC.

While all subtypes of epithelial ovarian cancer are worthy of attention, CCOC is the subtype with the worst 5-year survival rate for advanced-stage disease. The response to standard chemotherapy by patients with CCOC is less than 10% and in the recurrent setting is approximately 1% (27). The factors that contribute to the pathogenesis of CCOC or its poor response to therapy are ill-defined. Hence, we sought to extend our analysis of H2Bub1 in this histotype of ovarian cancer. Since RNF20/40 (5, 16, 20, 23, 28), ARID1A (18), and UBR7 (17) have been implicated in mediating H2B ubiquitination, we utilized IHC to characterize their expression in the aforementioned CCOC TMA. The cores were reviewed, scored, and compared as previously described.

**Figure 2:**
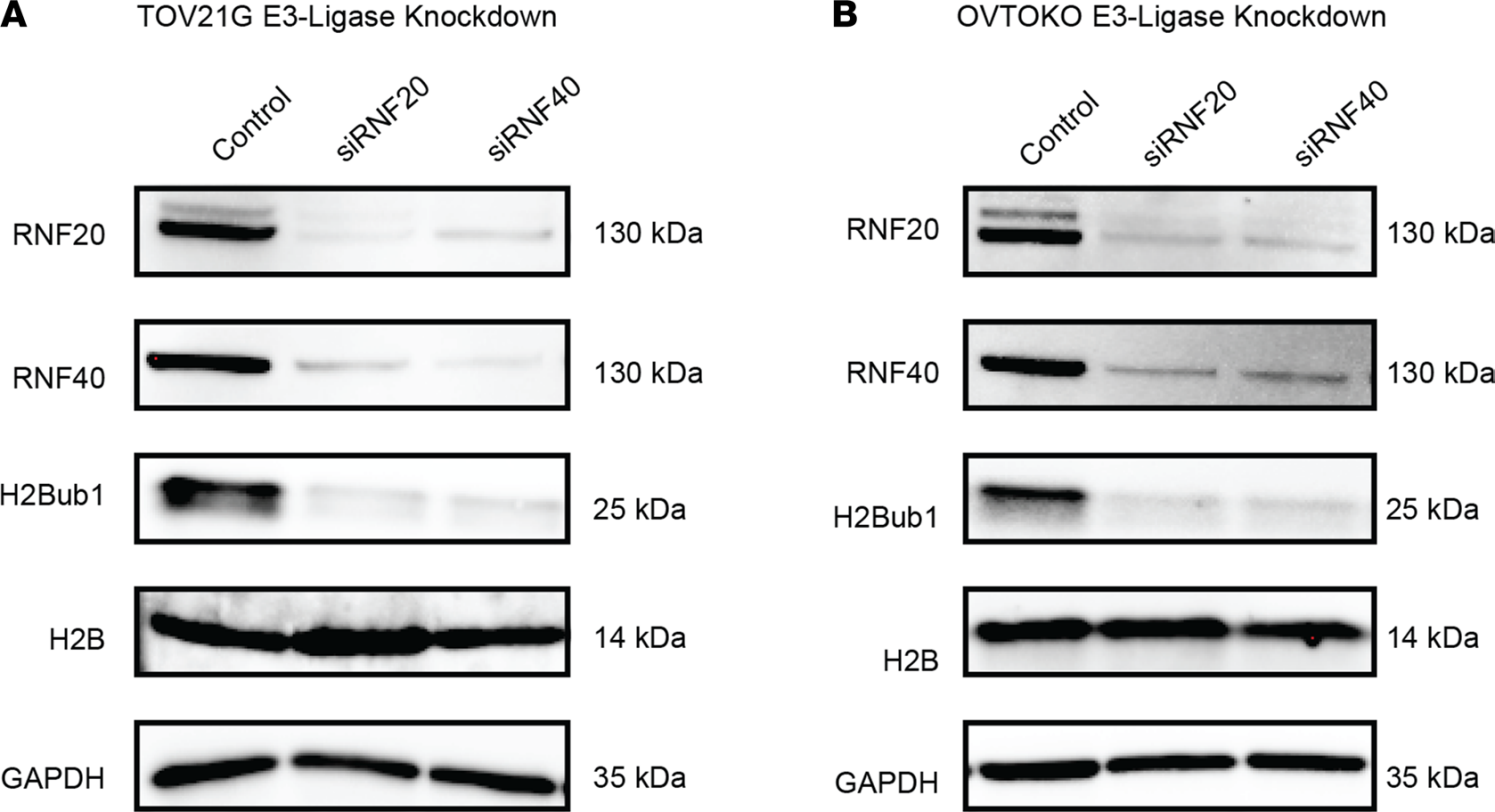
RNF20 and RNF40 are codependent in CCOC. siRNA-mediated knockdown of RNF20 and RNF40 in TOV21G (**A**) and OVTOKO (**B**) cell lines followed by Western blot analyses for protein expression with 1 representative loading control shown. Performed in triplicate.

**Figure 3:**
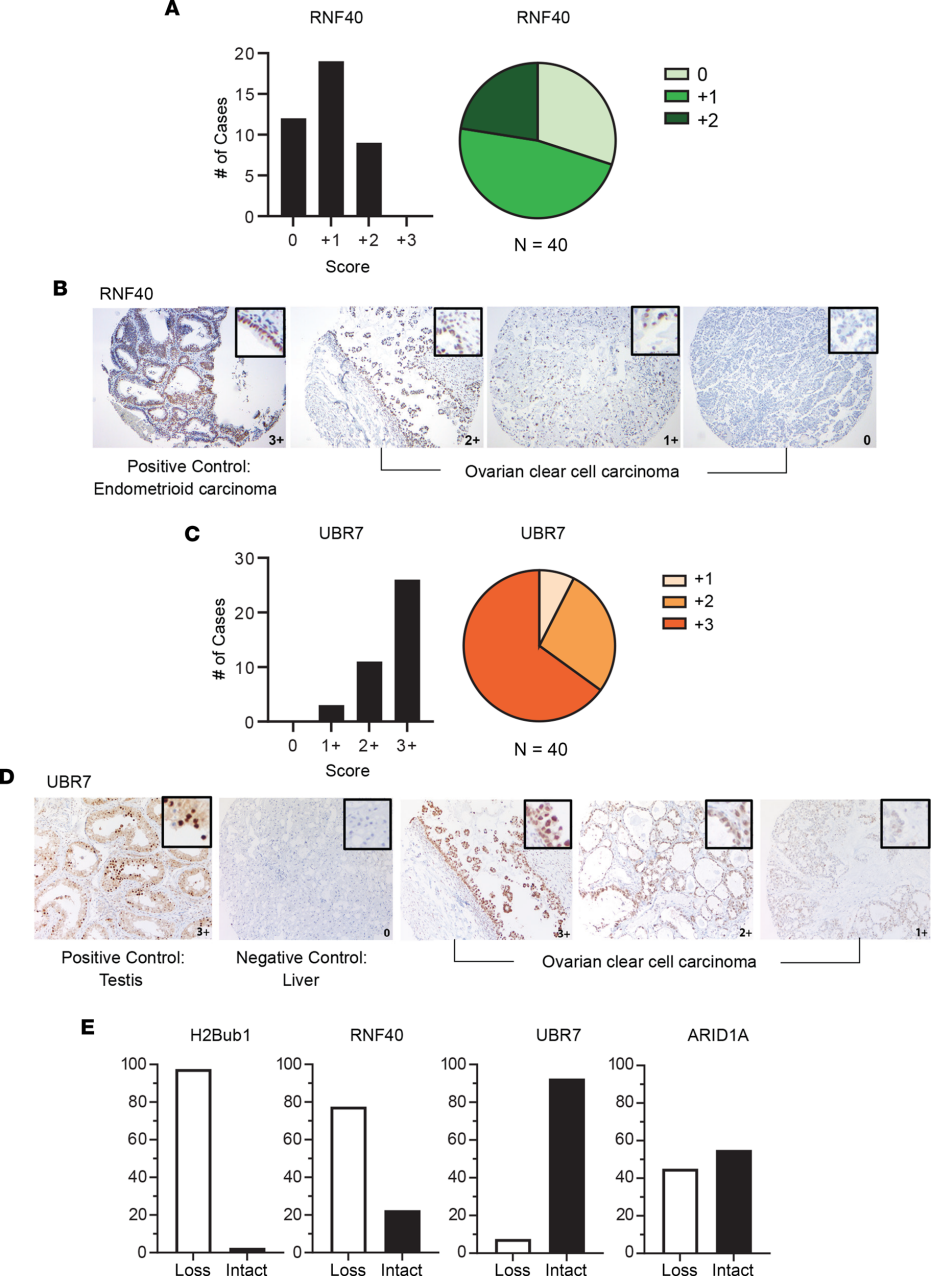
Drivers of H2Bub1 monoubiquitylation in CCOC. (**A** and **B**) Analysis of the E3 ligase RNF40 in a TMA of 40 cases with representative images. (**C** and **D**) Analysis of the ligase UBR7 among the same 40 cases with representative images. (**E**) Quantification after dichotomizing results for H2Bub1, RNF40, UBR7, and ARID1A. Samples that scored 0 or +1 CS are defined as “loss,” and samples that scored +2 or +3 CS are defined as “intact.”

To assess the expression of the RNF20/40 E3 ligase, we utilized antibodies against RNF40. The rationale for using antibodies against RNF40 is based on the reported mutual dependency between RNF20 and RNF40 for protein stability (16, 20, 23, 29). To assess whether this dependency is maintained in CCOC, we used RNA interference to independently knock down the expression of both RNF20 and RNF40 in 2 well-established CCOC cell lines, TOV21G and OVTOKO. Knockdown of either E3 ligase component led to the loss of the other and H2Bub1 (Figure 2, A and B; see complete unedited blots in the supplemental material). Hence, IHC for RNF40 is a clear surrogate for the expression of the RNF20/40 E3 ligase complex. Of the 3 E3 ligases examined, only RNF40, and thus the RNF20/40 complex, exhibited loss of expression at levels that paralleled the loss of H2Bub1. Specifically, 77.5% (31/40) of cases showed either low or negative staining for RNF40, which coincided with the loss of H2Bub1 (97.5%) in CCOC (Figure 3, A and B). In contrast, the majority of cases demonstrated positive expression of ligase UBR7 with 26/40 showing maximum intensity and percentage of cells (Figure 3, C and D). Approximately half of the cases (55%) showed loss of ARID1A expression (Figure 3E), consistent with the frequency of *ARID1A* mutation reported in the literature (18, 30, 31).

We extended our analysis by dichotomizing our CS into “loss” (0 or +1 CS) and “intact” (+2 or +3 CS), each defined by the binned value. Over three-quarters (77.5%) of cases were defined as loss for RNF40 and 98.5% for H2Bub1. ARID1A showed a moderate (55%) but less marked loss than RNF40. In contrast, the ligase UBR7 showed little (7.5%) loss in CCOC (Figure 3E).

In an effort to better understand the key regulator of H2Bub1 loss in cases of CCOC, we evaluated the status of each E3 ligase against levels of H2Bub1 in each case (Table 1). Approximately 80% (31/40) of cases with H2Bub1 loss showed low or no RNF40 expression. Importantly, we did not observe any cases that showed RNF40 loss with retained H2Bub1. In contrast, we observed a case with loss of ARID1A but with H2Bub1 retention. Furthermore, low ARID1A expression was only identified in 42.5% (17/40) of cases with absent H2Bub1 expression. Interestingly, we observed that 35% (14/40) of cases had dual loss of RNF40 and ARID1A, but this did not correlate with the extent of H2Bub1 loss. Levels of UBR7 did not correlate with the majority of H2Bub1-negative cases, with only 7.5% (3/40) of cases showing UBR7 loss (Table 1).

Our CS and case-by-case analyses supported the hypothesis that the loss of H2Bub1 in CCOC was driven primarily by the loss of the RNF20/40 E3 ligase complex and motivated us to further evaluate the relationship between this E3 ligase and H2Bub1. Using our CS analysis of the CCOC TMA, we examined whether low expression of RNF40 correlated with low H2Bub1. We compared average CS of each group and observed that H2Bub1 expression was significantly decreased compared with the core histone control H2B and UBR7 while levels of RNF40 were not statistically different from levels of H2Bub1 (Supplemental Figure 1A). Linear regression analysis showed a statistically significant positive correlation between H2Bub1 and RNF40 CS (*P* = 0.0003) based on the 95% CI of the slope (Supplemental Figure 1B). Similarly, H2Bub1 and RNF40 CS for each case ordered, by levels of H2Bub1, showed a positive trend with similar slopes for both CSs (Supplemental Figure 2A). Overall, these data suggest that those cases with low H2Bub1 expression also have low RNF40 expression, and as H2Bub1 levels increase, the levels of RNF40 follow (Supplemental Figure 2A).

### H2Bub1 loss is an early event in CCOC.

**Figure 4:**
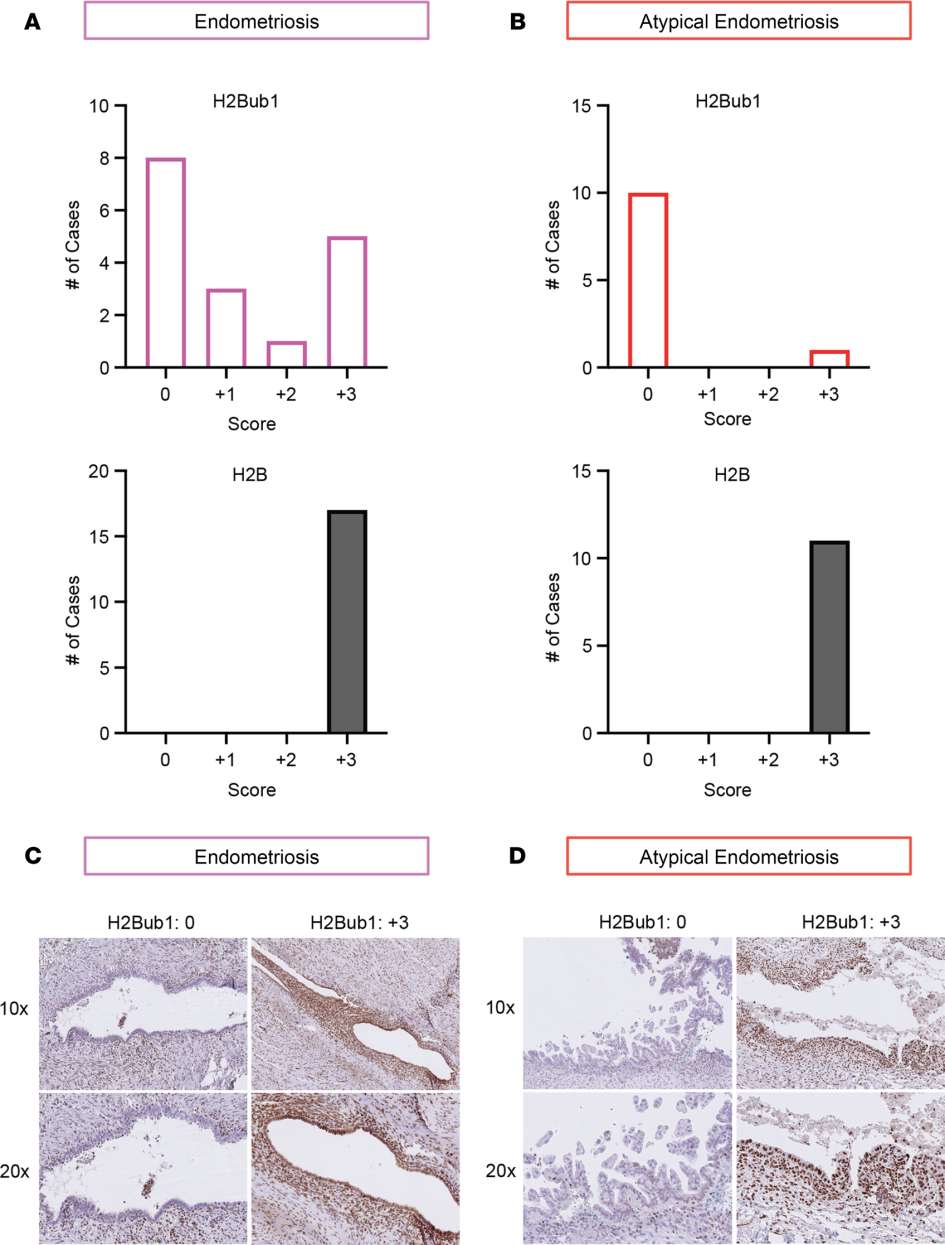
H2Bub1 loss is detected in CCOC precursors. (**A**) Analysis of both H2Bub1 and the core histone control, H2B, in 17 cases of endometriosis. (**B**) Analysis of both H2Bub1 and H2B in 11 cases of atypical endometriosis. (**C** and **D**) Representative images of positive and negative H2Bub1 staining in endometriosis and atypical endometriosis.

Work from our lab has traced the loss of H2Bub1 in HGSOC patient samples back to STIC lesions, making this an early event in tumor progression (20). In this study, the profound loss of H2Bub1 in CCOC motivated us to ask whether it is lost early in the pathogenesis of CCOC. Specifically, endometriosis and endometriotic lesions are known to be precursors to CCOC and therefore are important to understanding CCOC progression (24–27). To address this possibility, we characterized the levels of H2B and H2Bub1 in 17 cases of endometriosis and 11 cases of atypical endometriosis. Interestingly, in the setting of endometriosis, expression of H2Bub1 was low or negative in 65% (11/17) of cases (Figure 4A). Surprisingly, the loss of H2Bub1 was even more pronounced in atypical endometriotic lesions. We observed that all but a single case (10/11) of atypical endometriosis were negative for H2Bub1, translating to a 91% loss of H2Bub1 (Figure 4B). Representative images show a clear loss of H2Bub1 in the epithelial compartment of the lesions with retention in the stromal areas (Figure 4, C and D, and Supplemental Figure 3). The identification of H2Bub1 loss in CCOC precursors supports the assertion that it can be an early event in CCOC progression. Since endometriosis and atypical endometriosis are CCOC precursors of increasing severity (24–27) and H2Bub1 loss is more pronounced in atypical endometriosis, our data suggest that H2Bub1 loss may be necessary during transformation.

### DNA hypermethylation likely drives the loss of RNF40 and thus the depletion of H2Bub1.

Having demonstrated that H2Bub1 loss is strongly correlated with RNF20/40 deficit, we sought to determine the underlying mechanism affecting RNF20/40. We recently reported the role of DNA methylation in CCOC using genome-wide methylation analyses of 271 fresh-frozen tumor cases (32). While the analysis identified 2 equally sized clusters associated with distinct clinical features, it also afforded us the opportunity to determine whether hypermethylation of the *RNF40* gene may underlie the loss of the E3 ligase and H2Bub1.

**Figure 5:**
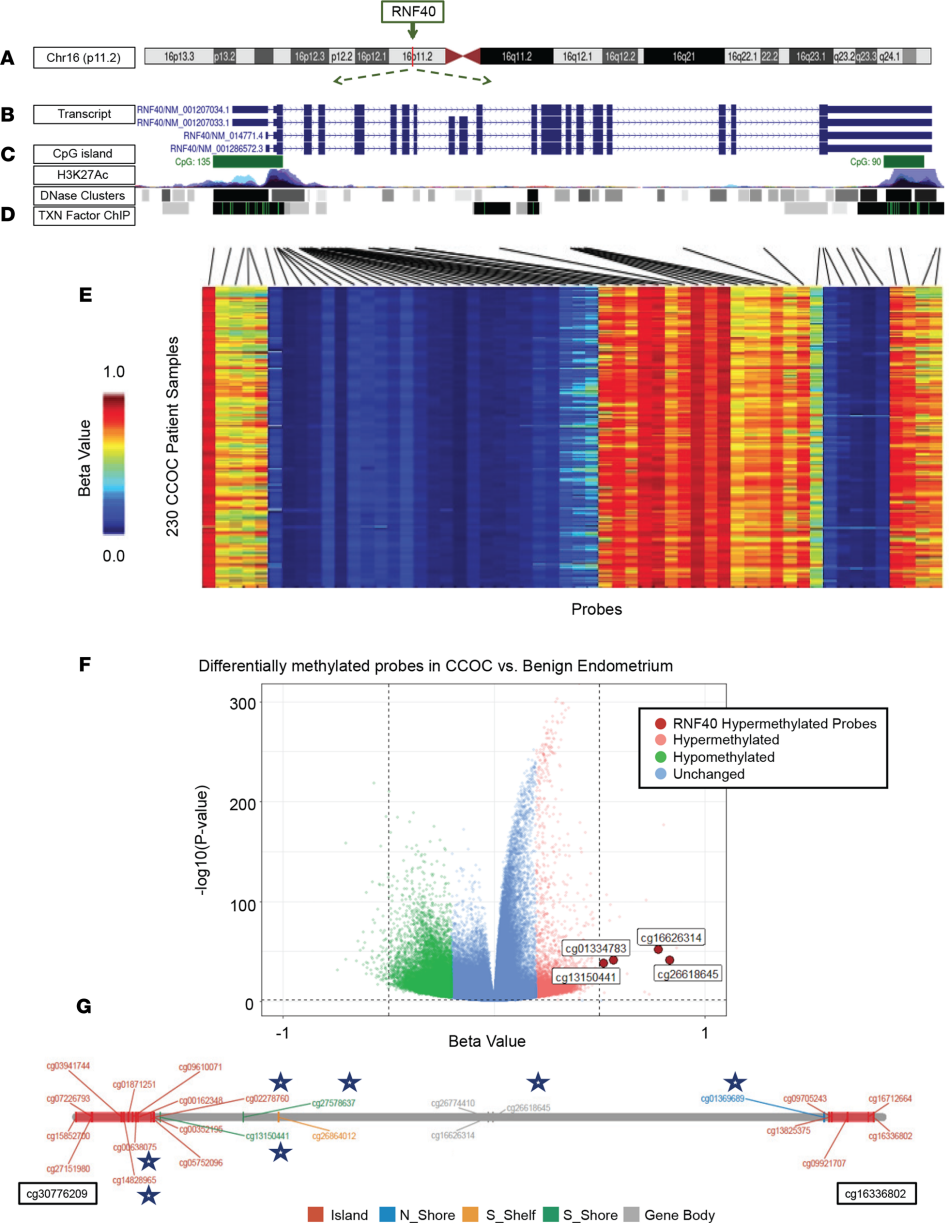
Mechanistic driver of H2Bub1 loss in CCOC. (**A**–**D**) UCSC genome browser schematic of the RNF40 gene encompassing chromogram, transcript, CpG islands, active enhancer marks (H3K27Ac), and transcription binding sites. (**E**) Heatmap showing β-values of RNF40 probes of methylation analysis for 230 CCOC patient samples; β-values of 0 depict hypomethylation and β-values of 1 signify hypermethylated probes. (**F**) Volcano plot comparing methylation status between benign endometrium and CCOC patient samples with an adjusted *P* value of less than 0.05 (horizontal dashed line) and Fisher’s FDR < 0.005. Hypermethylated probes are shown in pink, hypomethylated probes are shown by green, and RNF40 hypermethylated probes are highlighted in red with ID. Vertical dashed line represents –0.5 and 0.5 β-values. (**G**) Differentially methylated probes of the RNF40 locus in CCOC compared with endometriosis identified by blue star.

The *RNF40* gene is encoded on chromosome 16 (Figure 5, A and B). There are 2 CpG islands in *RNF40*: CpG island 135 in the promoter region and island 90 at the 3′ end of the gene (Figure 5C). Interestingly, the CpG island in the promoter region overlay prominent transcriptional binding sites and active enhancer marks (H3K27 acetylation; H3K27Ac) and may regulate *RNF40* expression (Figure 5, C and D). We noted a similar alignment of transcription factor binding sites and enhancer marks with the CpG island in the promoter of the *RNF20* locus on chromosome 9 (Supplemental Figure 4, A–D).

We then analyzed the DNA methylation status of all probes by plotting β-values, a quantitative measure of DNA methylation, with levels ranging from 0 (low; hypomethylated) to 1 (high; hypermethylated). In general, our analysis of *RNF40* showed that the methylation pattern of each probe was consistent across the 230 CCOC samples (Figure 5E). Notably, the probes overlying the CpG island near the promoter and transcription factor binding sites were strongly hypermethylated throughout all samples. We observed a similar trend in our analysis of *RNF20* (Supplemental Figure 4E). In addition, the 3′ end of *RNF40* containing the second CpG island also exhibited strong DNA hypermethylation (Figure 5E). These data identify DNA hypermethylation of CpG islands within the *RNF40* and *RNF20* loci that overlap with active enhancer marks (H3K27Ac) and transcription factor binding sites and likely control expression of these genes.

### The RNF40 gene is hypermethylated in CCOC compared with benign endometrium.

Motivated by our finding that H2Bub1 loss becomes more pronounced with disease progression (Figure 4), we compared the methylation status of *RNF40* between benign endometrium and CCOC. We analyzed a DNA methylation data set (33) that profiled the endometrial methylome, using the Illumina Infinium 450K panel, from 26 patients during different stages of their menstrual cycle, and compared the methylation levels of *RNF40* between cases of benign endometrium and CCOC. Our differential methylation analysis shows that *RNF40* was hypermethylated in CCOC compared with the tissue of origin, the endometrium, as represented in the volcano plot (Figure 5F). Specifically, *RNF40* was differentially hypermethylated in multiple locations within the promoter region, gene body, and 3′ untranslated region (Figure 5G). These data align with our finding that H2Bub1 loss occurs throughout CCOC progression and identify DNA hypermethylation of *RNF40* as a likely mechanism for these observations.

To experimentally test the connection between *RNF20/40* gene methylation and loss of H2Bub1 in CCOC, we analyzed a panel of 11 CCOC and fallopian tube cell lines for RNF20, RNF40, and H2Bub1 protein expression (Supplemental Figure 5; see complete unedited blots in the supplemental material). Our expectation was that some of these CCOC lines would mirror observations in tumor tissue and lack expression of the E3 ligase and H2Bub1. Surprisingly, all the lines had detectable levels of RNF20, RNF40, and H2Bub1. We suspected that this was attributable to the known effects of tissue culture on epigenetic changes, as recent studies have shown that in vitro culture can erode DNA methylation genome wide (34, 35). Accordingly, analysis of DNA methylation of *RNF20* and *RNF40* in the CCOC lines revealed that the promoter regions of both genes were grossly hypomethylated compared with the rest of the gene body (Supplemental Figures 6 and 7). These results support our hypothesis that DNA methylation is the underlying mechanism governing *RNF20* and *RNF40* expression in CCOC.

### H2Bub1 depletion contributes to transformation of CCOC precursors.

Thus far, we have shown that H2Bub1 is lost in the majority of CCOC cases and that DNA methylation at the *RNF40* and *RNF20* promoters is the likely underlying cause. Additionally, we have shown that H2Bub1 loss is an early event and becomes more pronounced during disease progression. However, it remains unclear whether loss of H2Bub1 functionally contributes to precursor transformation and tumorigenesis in CCOC. To assess the tumorigenic function of H2Bub1 loss in early stages of CCOC progression, we analyzed proliferation and clonogenicity in a known CCOC precursor cell line, 12Z, which is an immortalized human endometriotic cell line (36).

**Figure 6:**
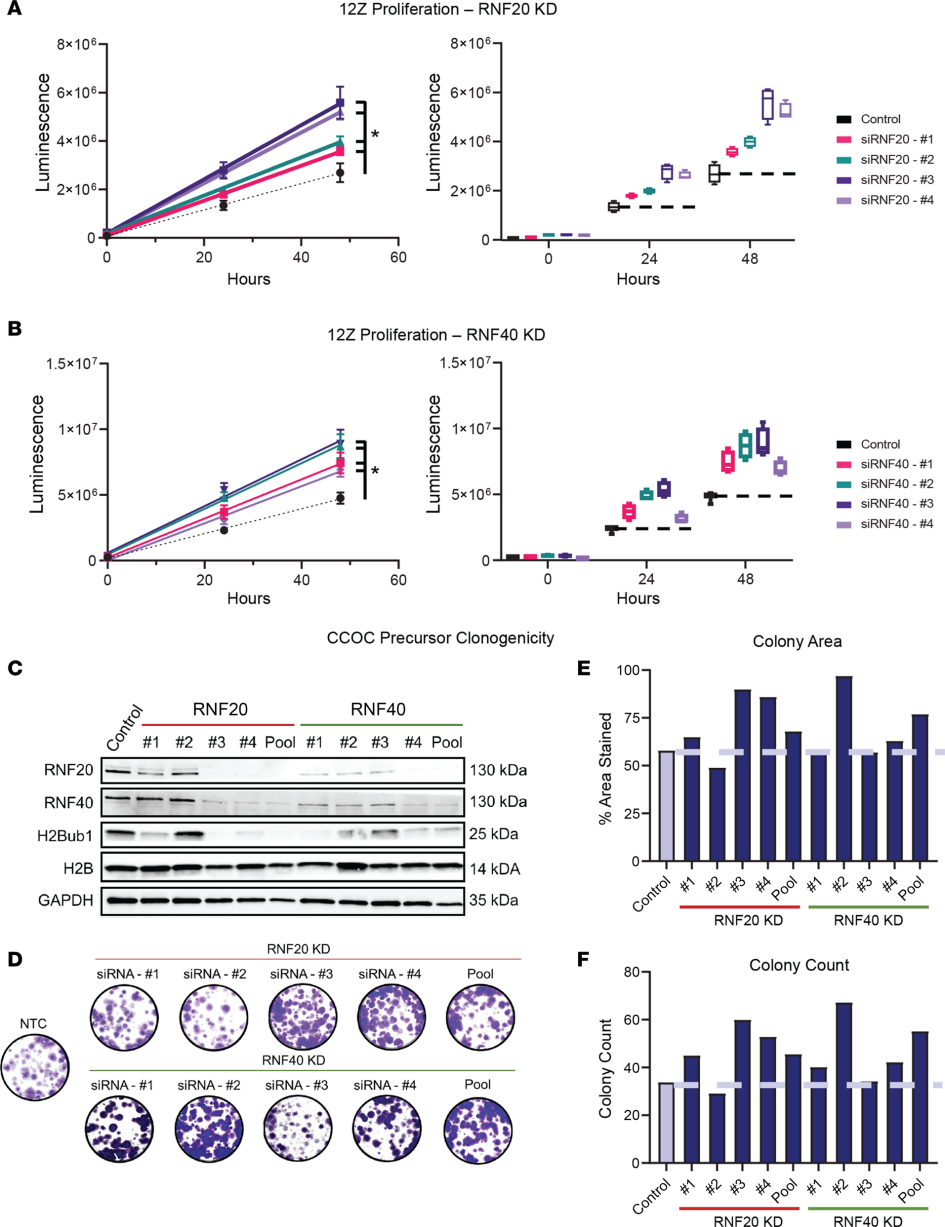
Functional consequence of H2Bub1 loss in CCOC precursors. (**A** and **B**) siRNA-mediated knockdown of RNF20 (**A**) and RNF40 (**B**) in 12Z cells followed by 48-hour proliferation analysis. Box plot (right) is a representation of scatterplot (left). Box plots show the interquartile range (box), median (line), and minimum and maximum (whiskers). *P* value of less than 0.05 represented by * (analyzed by Kruskal-Wallis 1-way ANOVA, followed by multiple pairwise comparisons using Dunn’s test). RNF20 and RNF40 knockdown in 12Z (**C**) followed by clonogenic analysis and quantification of colony area and colony count. Average of 2 biological replicates (**D**–**F**). Representative images of entire well shown. NTC, nontargeting control.

To analyze the effects of H2Bub1 depletion on cell proliferation, we knocked down RNF20 and RNF40 in 12Z using 4 siRNAs each. All knockdowns for both RNF20 and RNF40 significantly increased proliferation over 48 hours compared with the control group (Figure 6, A and B, and Supplemental Figure 8, A and B; see complete unedited blots in the supplemental material). In addition, a clonogenicity assay was performed, with either RNF20 or RNF40 knockdown confirmed by Western blot (Figure 6, C and D). Both knockdowns of RNF20 and RNF40 increased colony area and colony count (Figure 6, D–F). As expected, the clonogenic effects aligned with the degree of protein knockdown (Figure 6, C–F). As H2Bub1 loss increased proliferation and clonogenic growth in CCOC precursors, these data suggest that the expression of the RNF20/40 complex and thus the presence of H2Bub1 serves as a brake to neoplastic growth and that its loss heralds a more aggressive phenotype in the progression of CCOC.

## Discussion

In this study, we report on the expression, mechanism, and function of H2Bub1 in histotypes of epithelial ovarian cancer. We use the combination of primary patient tissues, large whole-genome data sets, and in vitro biochemistry to make 5 potentially novel observations. First, we used IHC to show that expression of H2Bub1 is profoundly lost in CCOC while its loss in LGSOC and endometrioid cancers is less severe. Second, we showed that loss of the RNF20/40 E3 ligase complex strongly correlated with H2Bub1 loss, while the expression of other E3 ligases did not. Third, we determined that DNA hypermethylation of the RNF20/40 complex is the underlying mechanism likely driving RNF20/40 loss and thus H2Bub1 depletion in CCOC. We further show that H2Bub1 becomes progressively lost from endometriosis to atypical endometriosis and finally to CCOC. The loss of H2Bub1 tracks with *RNF40* DNA hypermethylation. Finally, we show that the depletion of both RNF20 and RNF40, and thus H2Bub1, in CCOC precursors has functional consequences and leads to early-stage transformation and CCOC progression.

The data presented here support the hypothesis that H2Bub1 represents an important roadblock to neoplastic transformation in CCOC progression. This is consistent with work in other solid tumors, including colorectal cancer, lung adenocarcinoma, and HGSOC (15, 19, 20, 23). In our analysis of H2Bub1 in CCOC, LGSOC, and endometrioid cancer, we found that each histotype exhibited some degree of H2Bub1 loss, suggesting that H2Bub1 loss is a common event in all epithelial ovarian cancer histotypes. However, CCOC showed the most significant loss as the majority of cases were negative for H2Bub1 (97.5%). This degree of loss surpassed the proportion found in HGSOC (5, 19, 20). Together with the fact that CCOC is an aggressive and typically therapy-resistant tumor type, these data motivated us to further explore the role of H2Bub1 in the development of this histotype. Endometriosis and atypical endometriosis are known precursors to CCOC (24–27). Our analysis of these precursors showed that H2Bub1 loss is an early event, escalates throughout CCOC pathogenesis, and functionally contributes to neoplastic transformation in this setting. This suggests that H2Bub1 status is a key component in precursor stage transition and CCOC progression. Given its role in chromatin compaction and gene expression, pathways that are deregulated by H2Bub1 may represent early-stage biomarkers or therapeutic targets of endometriosis and CCOC.

Epigenetic changes are considered to be among the earliest genomic aberrations during tumorigenesis, including in HGSOC (2, 3). We sought to determine whether loss of DNA methylation at the RNF40 locus could account for the deficiency of H2Bub1 observed in CCOC. Specifically, a large multi-institutional sequencing effort on CCOC patient samples (32, 37) has established a TCGA-like database for facilitating CCOC translational research. Utilizing the genome-wide DNA methylation profiles from these studies, we determined that the *RNF40* gene is hypermethylated in CCOC. Interestingly, *RNF20* expression has previously been shown to be suppressed by promoter hypermethylation in breast cancers (38), making its reactivation and the restoration of H2Bub1 possible through the use of DNA methylation inhibitors. Of interest in our study, hypermethylation of *RNF40* occurs within key areas of gene regulation, specifically CpG islands in promoter regions overlying transcriptional binding sites and active enhancer marks (H3K27Ac). Methylation in these areas is known to decrease transcription initiation and silence gene expression. A temporal analysis of *RNF40* methylation in CCOC compared with benign endometrial tissue showed that *RNF40* is hypermethylated in the invasive disease when compared with endometrium. Together with our IHC data of RNF40 and H2Bub1, case-by-case observation, and statistical correlation, the hypermethylation of *RNF40* and differential methylation temporally both align with and provide a mechanistic understanding of what drives the loss of H2Bub1 in CCOC patients and disease progression. This effort provides insights that may benefit future therapeutic studies targeting the methylome or noninvasive early cancer detection approaches measuring chromatin changes in cell-free DNA (39).

Understanding the mechanism of RNF40 loss, and thus the depletion of H2Bub1, led us to inquire why this occurs and what benefit it confers on CCOC precursor lesions. Biochemical analysis of an immortalized human endometriotic epithelial cell line, 12Z (36), provided an opportunity to characterize the functional consequences of H2Bub1 loss. Knockdown of RNF20/40 (with concomitant H2Bub1 reduction) led to a more aggressive phenotype as measured by cell proliferation and clonogenic growth.

Our study is the first, to our knowledge, reporting on the state of H2Bub1 in various subtypes of ovarian epithelial carcinomas and determining the temporal status within CCOC progression. We believe we are also the first to analyze its relevant E3 ligases in CCOC and resolve the associated mechanism likely driving this epigenetic aberration in the context of ovarian cancer. These data highlight the significance of H2Bub1 in CCOC and point toward opportunities for early detection and therapeutic intervention.

## Methods

### TMA.

CCOC and LGSOC TMAs were constructed from 40 and 44 cases, respectively, from patients who underwent cytoreductive surgery at the Hospital of the University of Pennsylvania or at the University of Pittsburgh Medical Center. Hematoxylin and eosin–stained slides were reviewed prior to TMA construction by 3 pathologists to verify the diagnosis and the presence of sufficient tissue. Each case was represented by triplicate cores, 0.8 and 2.0 mm in diameter, respectively. Eighteen cases of endometrioid carcinoma obtained from the Hospital of the University of Pennsylvania were also used. Slides were immunostained for H2B, H2Bub1, RNF40, ARID1A, and UBR7 separately, and each core was scored as described in *IHC scoring*. See Supplemental Table 3 for antibody conditions. In addition, 17 cases of endometriosis and 11 cases of atypical endometriosis were obtained, reviewed, and stained for H2Bub1 and H2B.

### IHC.

IHC staining was performed using EnVision+/Horseradish Peroxidase system (DAKO). Formalin-fixed, paraffin-embedded tissue sections were dewaxed, rehydrated, and incubated in hydrogen peroxide solution for 30 minutes to block endogenous peroxidase activity. Antigen retrieval was carried out by pressure cooker treatment in citrate buffer (pH 6.0) for 40 minutes. Sections were incubated with primary antibody using the conditions specified in Supplemental Table 3. Secondary antibody (DAKO EnVision+ System-HRP Labelled Polymer anti-mouse, catalog K4001) was applied for 30 minutes, followed by DAB for 5 minutes. Positive controls included breast cancer tissue, fallopian tube, testis, and endometrioid carcinoma.

### IHC scoring.

The ovarian CCOC TMA, LGSOC TMA, and endometrioid carcinoma whole-mount tissue slides were stained for H2Bub1, RNF40, H2B, ARID1A, and UBR7; reviewed; and scored semiquantitatively for staining intensity (SI) and percentage of positive cells (PP) by up to 6 pathologists. Additionally, whole-mount tissue slides of endometriosis and atypical endometriosis were also stained for H2B and H2Bub1. PP was scored as follows: 0 (<5% cells positive), 1 (5%−9% cells positive), 2 (10%−74% cells positive), 3 (75%−100% cells positive). SI was assessed as follows: 0 (negative), 1 (weak), 2 (moderate), 3 (strong). Subsequently, a CS for each case was calculated by multiplying the PP score of 0−3 by the corresponding SI score of 0−3. Ultimately, the expression level of each marker was defined based on the CS: 0 (for CS ≤ 1), 1+ (for 1 < CS ≤ 3), 2+ (for 3 < CS ≤ 5), and 3+ (for CS > 5). Benign tissues including ovary, fallopian tube, endometrium, testis, breast, kidney, and liver served as controls and were included in each TMA and assessed for every antibody.

### Cell culture and gene silencing.

All cell lines were authenticated using short tandem repeat profiling within the last 4 months (IDEXX). All experiments were performed with mycoplasma-free cells, confirmed by the Cambrex MycoAlert assay at the University of Pennsylvania School of Medicine Cell Center. Ten human clear cell carcinoma cell lines were used in these studies. ES-2 (RRID:CVCL_3509), TOV-21G (RRID:CVCL_3613), OVTOKO (RRID:CVCL_3117), OVMANA (RRID:CVCL_3111), and OVKATE (RRID:CVCL_3110) were obtained from the American Tissue Type Collection and as a gift from Gottfried Konecny (UCLA, Los Angeles, California, USA). OCI-C5x (RRID:CVCL_DH06) was a gift from Tan A. Ince (University of Miami, Miami, Florida, USA). JHOC-5 (RRID:CVCL_4640), JHOC-7 (RRID:CVCL_4641), and JHOC-9 (RRID:CVCL_4643) were purchased from RIKEN BioResource Center. OVISE (RRID:CVCL_3116) was a gift from David Huntsman (The University of British Columbia, Vancouver, British Columbia, Canada). 12Z (RRID:CVCL_0Q73) endometriosis cells were a gift from Rugang Zhang (The Wistar Institute, Philadelphia, Pennsylvania, USA). In addition, 2 fallopian tube epithelium cell lines, FT246 (RRID:CVCL_UH61) and hTERT FT282 (RRID:CVCL_A4AX), were developed in the lab and previously described (40, 41). Cells were cultured in a 5% CO_2_ atmosphere at 37°C in their respective media. OVTOKO, OVMANA, OVISE, OVKATE, and 12Z were cultured in RPMI 1640 (Thermo Fisher Scientific) medium with 10% fetal bovine serum (FBS; Atlanta Biologicals) and supplemented with 1% antibiotics. JHOC-5, JHOC-7, and JHOC-9 were cultured in DMEM/F12 1:1 (Cellgro) with 10% FBS supplemented with 0.1 mM nonessential amino acids and 1% penicillin/streptomycin. ES-2 was cultured in McCoy’s 5a media supplemented with 10% FBS and 1% antibiotics. TOV21G was cultured in 1:1 mixture of MCDB105 and Medium 199 supplemented with 10% FBS and 1% antibiotics. OCI-C5X was cultured in OCMI-e media (USBiological) supplemented with 2% FBS and 25 ng/mL of cholera toxin without azide. Fallopian tube lines were cultured in DMEM/F12 1:1 (Cellgro) with 2% Ultroser G serum substitute (Pall Life Science).

RNF20 and RNF40 knockdown was performed by RNA interference using siRNA oligo transfection. siRNA knockdown was achieved with Dharmafect 1 reagents according to the manufacturer’s protocol (Dharmacon RNAi technologies). siRNA oligonucleotides, described in Supplemental Table 4, were purchased from Dharmacon. The final concentration of all siRNA oligonucleotides was 25 nM. Cells were washed twice with PBS, harvested, centrifuged for 3 minutes at 123*g*, and plated for assays and/or frozen at –80 °C until lysis.

Cell proliferation assay was performed in triplicate using CellTiter-Glo luminescence reagent (Promega) following the manufacturer’s guidelines. Briefly, 5,000 cells were seeded in 96-well plates in 100 mL of the media with CellTiter-Glo reagent, then allowed to grow for various time intervals, and luminescence was recorded. Clonogenic assays were also performed in triplicate by seeding cells in a 6-well plate at 500 cells and incubating for 14 days. Cells were fixed with 4% paraformaldehyde in PBS, then stained with 0.5% crystal violet, and images were captured.

### Bioinformatics and statistics of DNA methylation.

We analyzed CCOC methylation data from patient samples obtained from NCBI Gene Expression Omnibus GSE185008 (32) with 230 samples. For comparative analysis with precursor tissues, endometrial methylome data from patients with endometriosis was obtained from a published study (33) and subseries NCBI Gene Expression Omnibus GSE73948 with 26 samples. The data were preprocessed by using minfi R package v1.42.0. (42). Quantile normalization was done by using preprocess Quantile function of the minfi package, and β-values were calculated to quantify the level of methylation. SeSAMe (43) package was used to plot heatmaps for the *RNF40* gene. To identify differentially methylated probes between CCOC and endometriosis, we used the function dmpFinder in the minfi package to obtain *t* statistics and associated *P* values for each probe.

OVTOKO, OVMANA, TOV21G, JHOC-5, JHOC-7, and JHOC-9 cell lines were analyzed using the Illumina EPIC or 450K arrays (35). Raw IDAT files were processed and normalized, and β-values were calculated as previously described (35). Probes annotated to *RNF20* or *RNF40* were selected for further analyses (Supplemental Figures 8 and 9).

### Western blot analysis.

Samples were lysed using RIPA buffer supplemented with protease and phosphatase inhibitor (Thermo Fisher Scientific). Samples were sonicated as described (44). Protein content of whole-cell lysates was quantified using Pierce BCA kit protocol (Thermo Fisher Scientific). Proteins were separated on a 4%–15% gradient SDS-PAGE (Bio-Rad) before being transferred to a PVDF membrane using the Turbo Blot system (Bio-Rad). Membranes were incubated with primary antibody overnight at 4°C (Supplemental Table 3). After washing, membranes were incubated with an anti-rabbit HRP-conjugated secondary antibody for 1 hour. Proteins were detected using Pico PLUS Chemiluminescent substrate (Thermo Fisher Scientific) and visualized with a ChemiDoc imaging system (Bio-Rad).

### Statistics.

Statistical analysis was performed using Prism 9.1.2 (GraphPad Software Inc.) and Origin Pro software. Nonparametric testing of average composite scores between the groups was analyzed by Kruskal-Wallis 1-way ANOVA, followed by multiple pairwise comparisons using Dunn’s test with *P* value of <0.05 determining significant differences among the groups. Significant differences with a *P* value of <0.05 are identified with *. CS correlation between H2Bub1 and RNF40 was determined by linear regression analysis including linear slopes and positive/negative 95% CI.

### Study approval.

The Institutional Review Boards at the University of Pennsylvania and the University of Pittsburgh Medical Center, Pittsburgh, Pennsylvania, USA, approved the study protocol, and the deidentified tissue blocks were obtained from the Department of Pathology at both institutions with written informed consent.

## Author contributions

AJF and RD conceived and designed the study; KD, XZ, AB, JLT, MA Morgan, TRS, and LES coordinated tissue acquisition; AJF, HSR, YK, and MA Mitchell performed experiments; AVA, RBS, and VEV performed acquisition of DNA methylation data; AJF, PR, HSR, AVA, XZ, AB, RBS, TRS, VEV, and RD performed analysis and interpretation of data. AJF, AVA, PR, HSR, MA Mitchell, RBS, VEV, and RD wrote, reviewed, and revised the manuscript. RD supervised the study. The work reported in the paper has been performed by the authors, unless clearly specified in the text.

## Supplementary Material

Supplemental data

## Figures and Tables

**Table 1:**
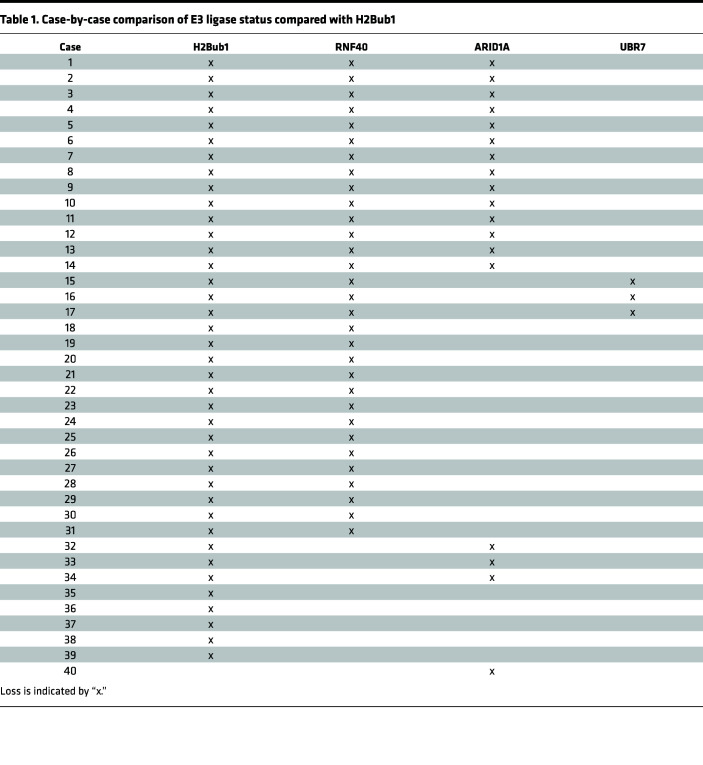
Case-by-case comparison of E3 ligase status compared with H2Bub1

## Version 1 06/22/2023

Electronic publication
