# Supplementary material for: H2Bub1 loss is an early contributor to clear cell ovarian cancer progression
Source: JCI Insight. 2023 Jun 22;8(12):e164995. doi: 10.1172/jci.insight.164995 (PMC10371241; doi:10.1172/jci.insight.164995)
Supplement: Supplemental data [file jciinsight-8-164995-s114.pdf]

Supplemental Data

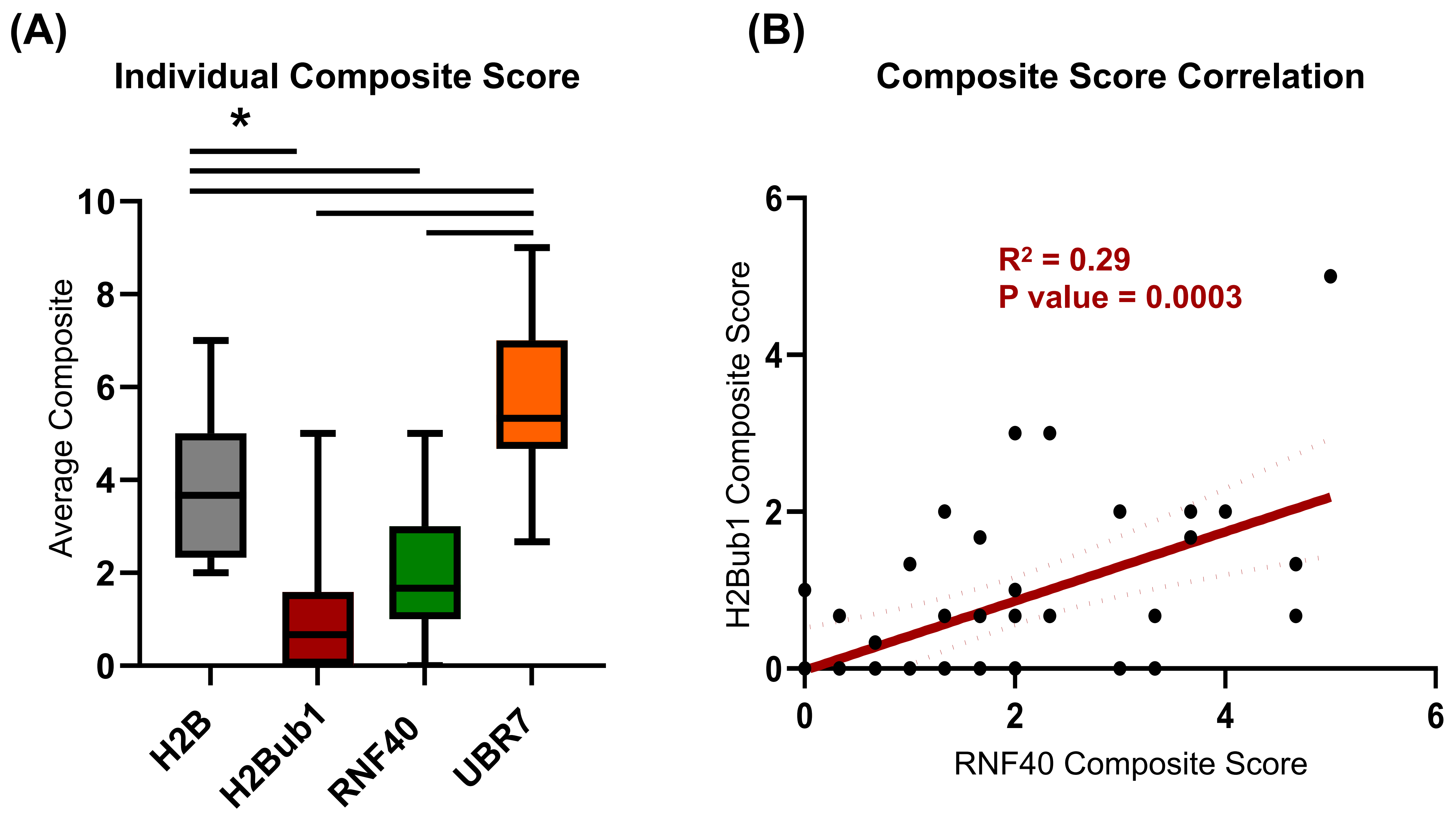

Supplemental Figure 1. Statistical correlation between H2bub1 loss and RNF40. (A) Comparison of composite scores for H2B, H2Bub1, RNF40, and UBR7. P value of <0.05 represented by *. Each solid black line represents comparisons with P value of <0.05 and noted with the single * (analyzed by Kruskal-Wallis ANOVA, followed by multiple pairwise comparisons using Dunn's test) (B) Pearson correlation analysis between H2Bub1 and RNF40 composite scores. Dashed lines are the 95% confidence threshold.

(A)

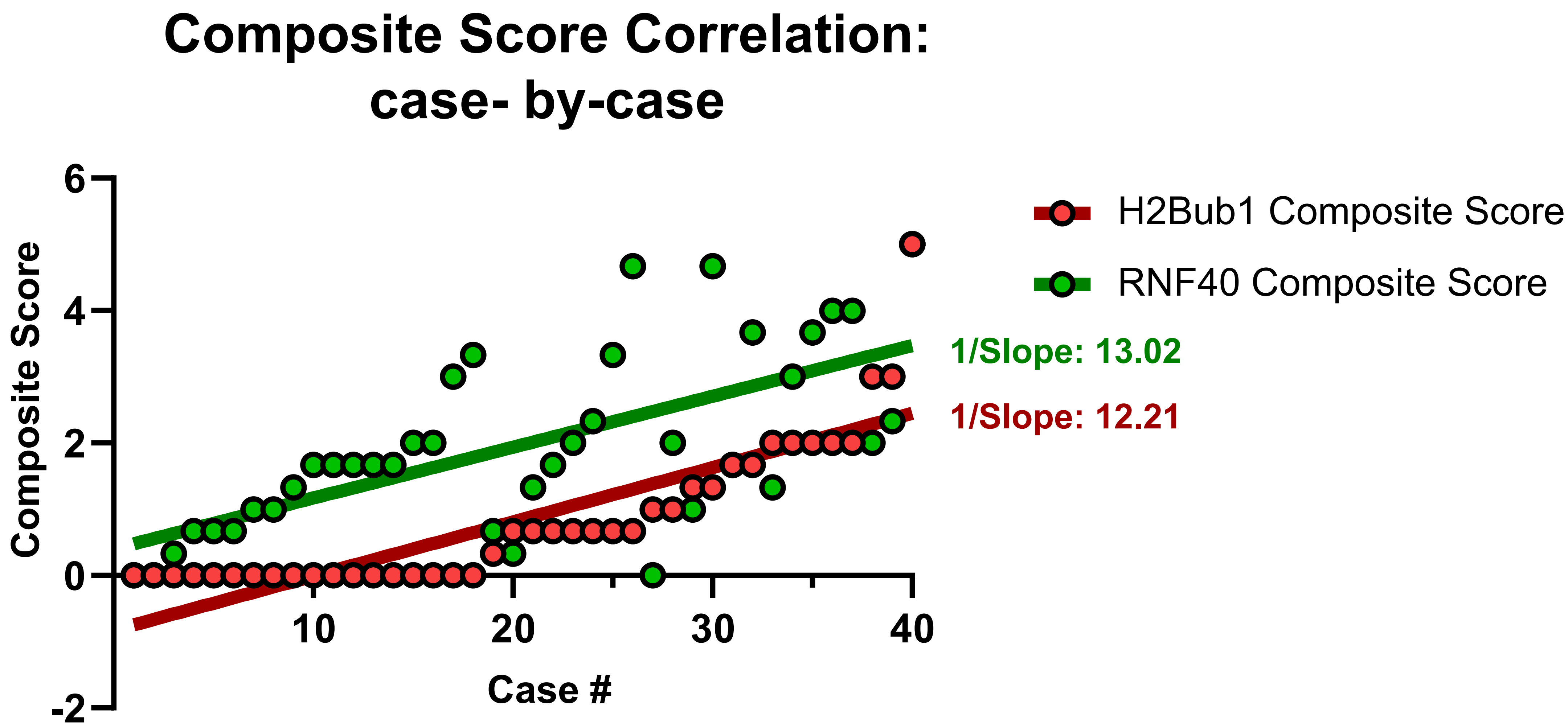

Supplemental Figure 2. Case-by-case composite analysis. (A) Comparison of composite scores of H2Bub1 and RNF40 case-by-case. Linear regression analysis of each composite score, 1/slope shown on graph.

(A)

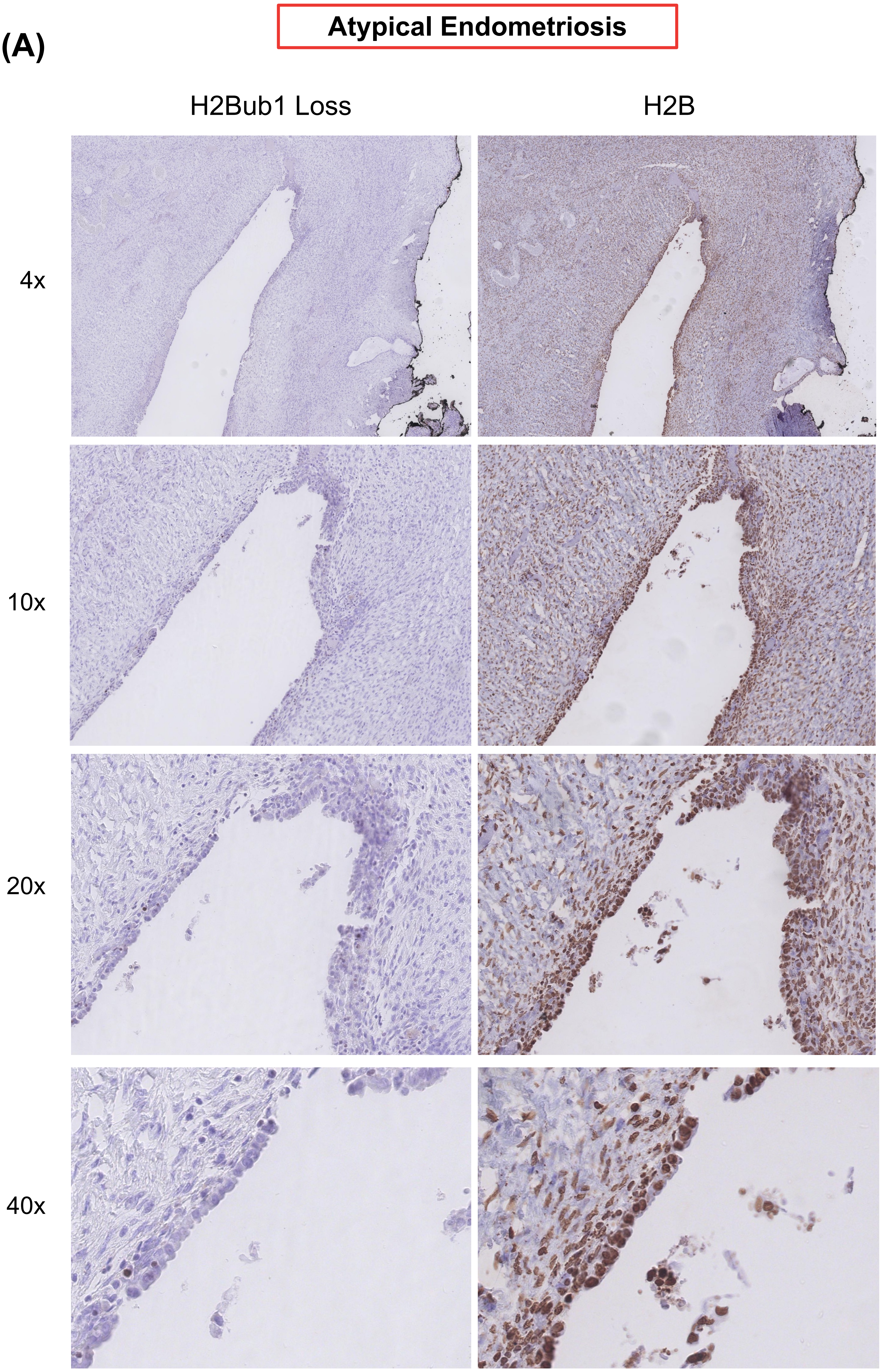

Supplemental Figure 3. Additional images. (A) Atypical endometriosis cases with H2Bub1 loss or H2B at 4x, 10x, 20x, and 40x magnification.

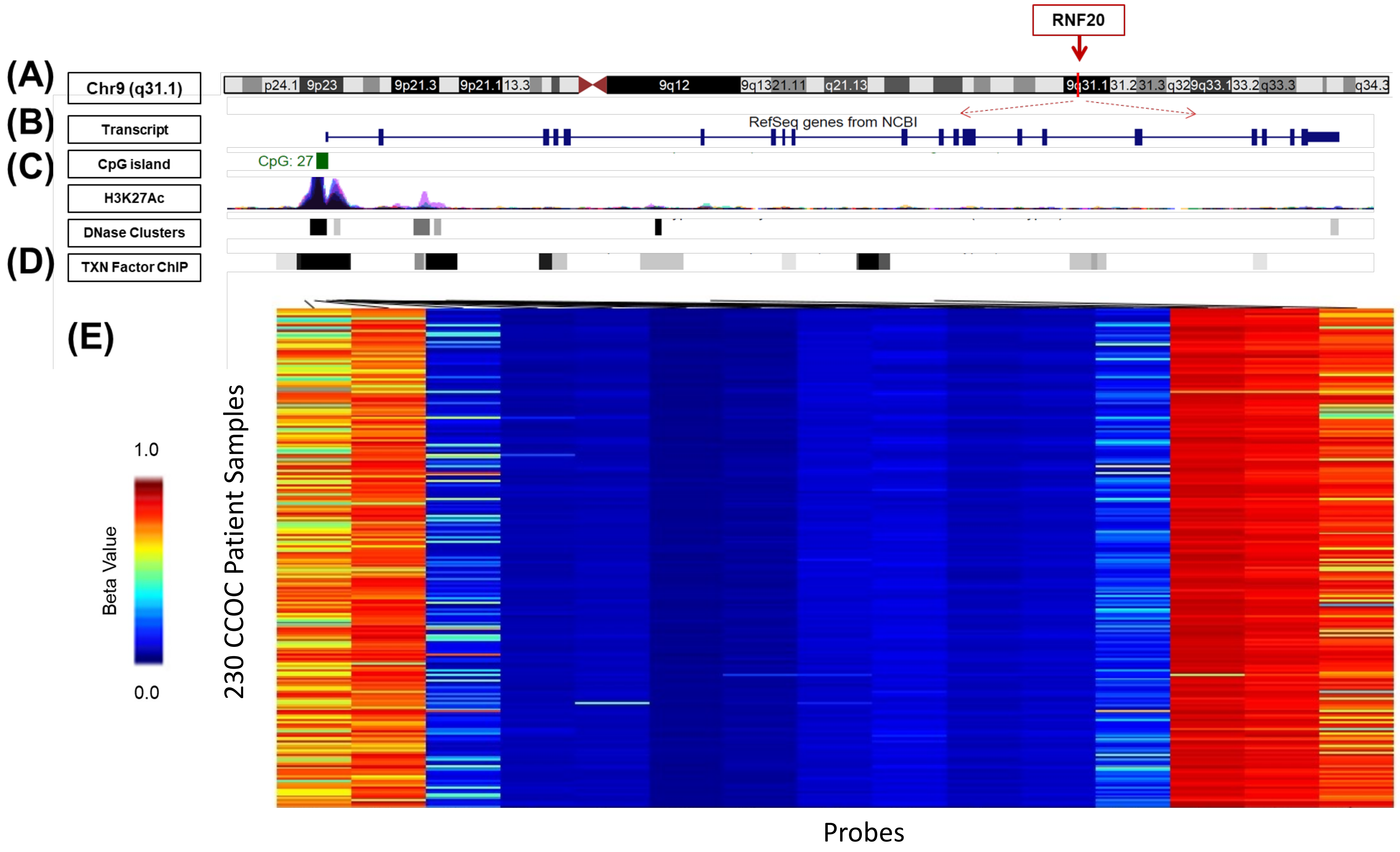

Supplemental Figure 4. Methylation analysis of *RNF20*. (A-D) UCSC genome browser schematic of the *RNF20* gene encompassing chromogram, transcript, CpG islands, active enhancer marks (H3K27Ac), and transcription binding sites. (E) Heatmap showing beta values of *RNF20* probes of methylation analysis for 230 CCOC patient samples. Beta values of 0 depict hypomethylation and beta values of 1 signify hypermethylated probes.

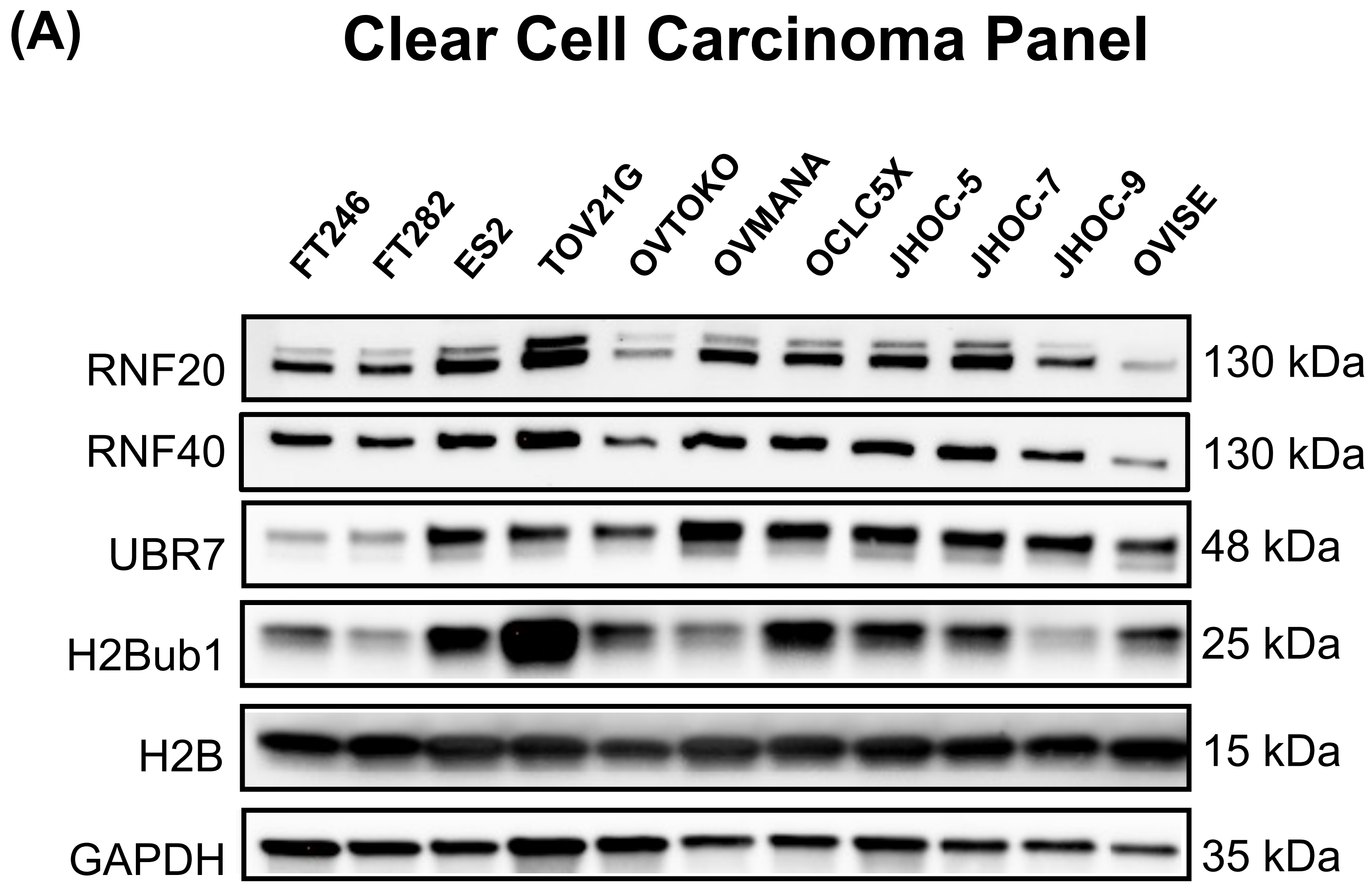

Supplemental Figure 5. CCOC panel analysis. (A) Western blot of 8 established CCOC cell lines and two fallopian tube secretory epithelial cells, FT246 and FT282. H2B and GAPDH serve as loading controls. Performed in duplicate.

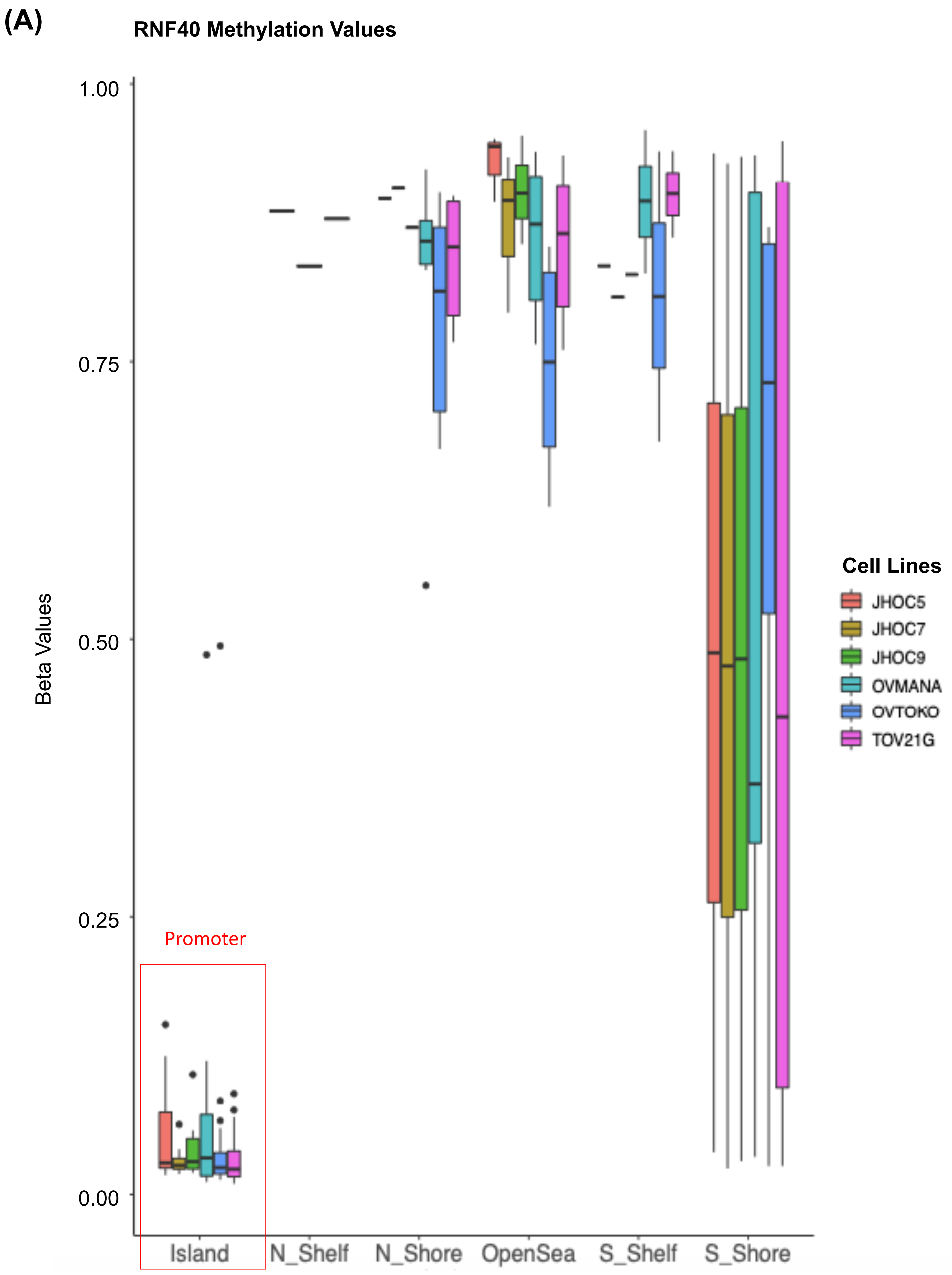

Supplemental Figure 6. DNA methylation analysis of CCOC cell lines. (A) Boxplot showing beta values from methylation analysis of CpG surrounding *RNF40* in regions of Islands, North Shelf, North Shore, Open Sea, South Shelf, and South Shore in six CCOC cell lines: JHOC5, JHOC7, JHOC9, OVMANA, TOV21G, and OVTOKO. Smaller Beta values depict unmethylated CpG and larger beta values signify methylated CpG. Red box identifies promoter region.

(A)

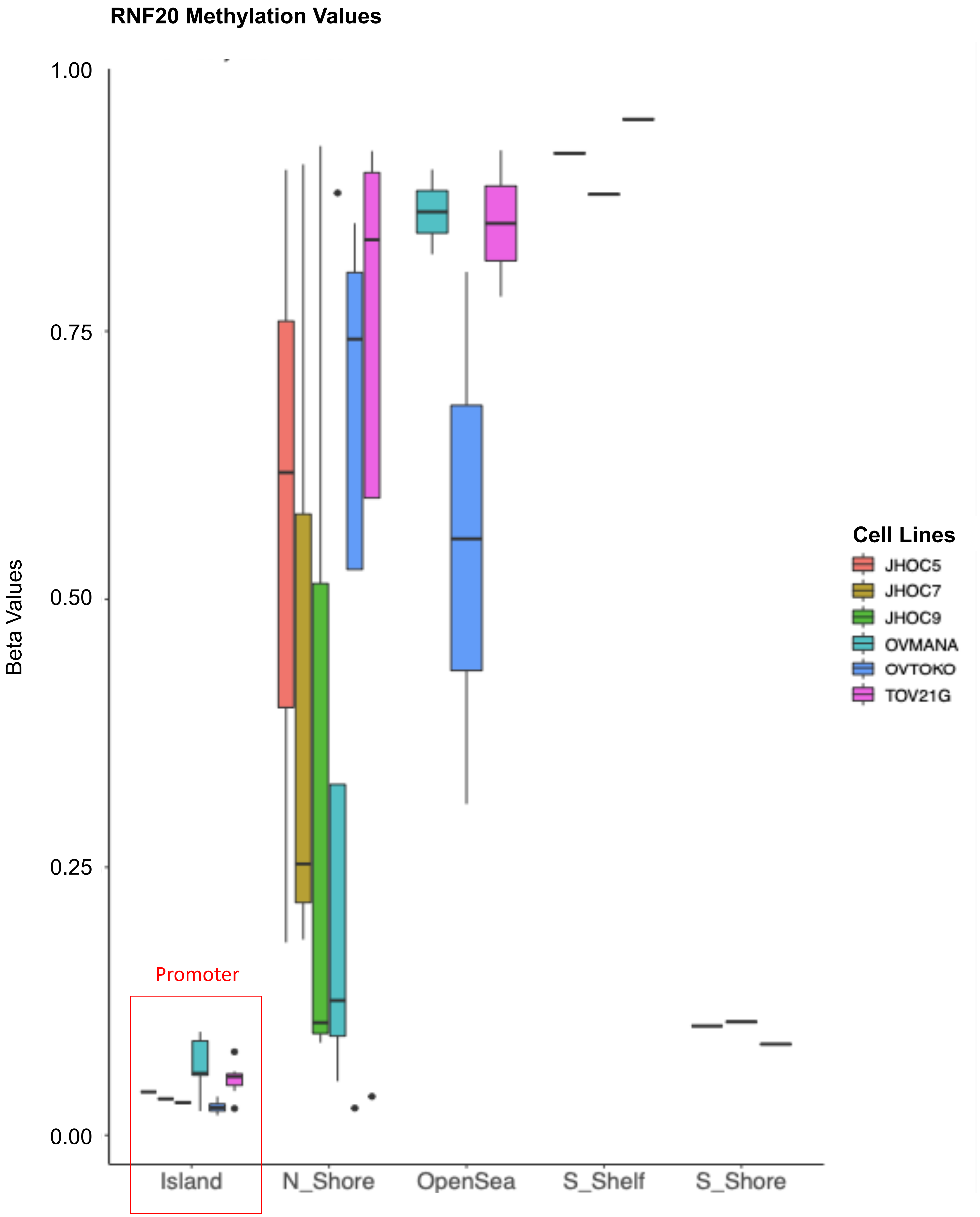

Supplemental Figure 7. DNA methylation analysis of CCOC cell lines. (A) Boxplot showing beta values from methylation analysis of CpG surrounding *RNF20* in regions of Islands, North Shelf, North Shore, Open Sea, South Shelf, and South Shore in six CCOC cell lines: JHOC5, JHOC7, JHOC9, OVMANA, TOV21G, and OVTOKO. Smaller Beta values depict unmethylated CpG and larger beta values signify methylated CpG. Red box identifies promoter region.

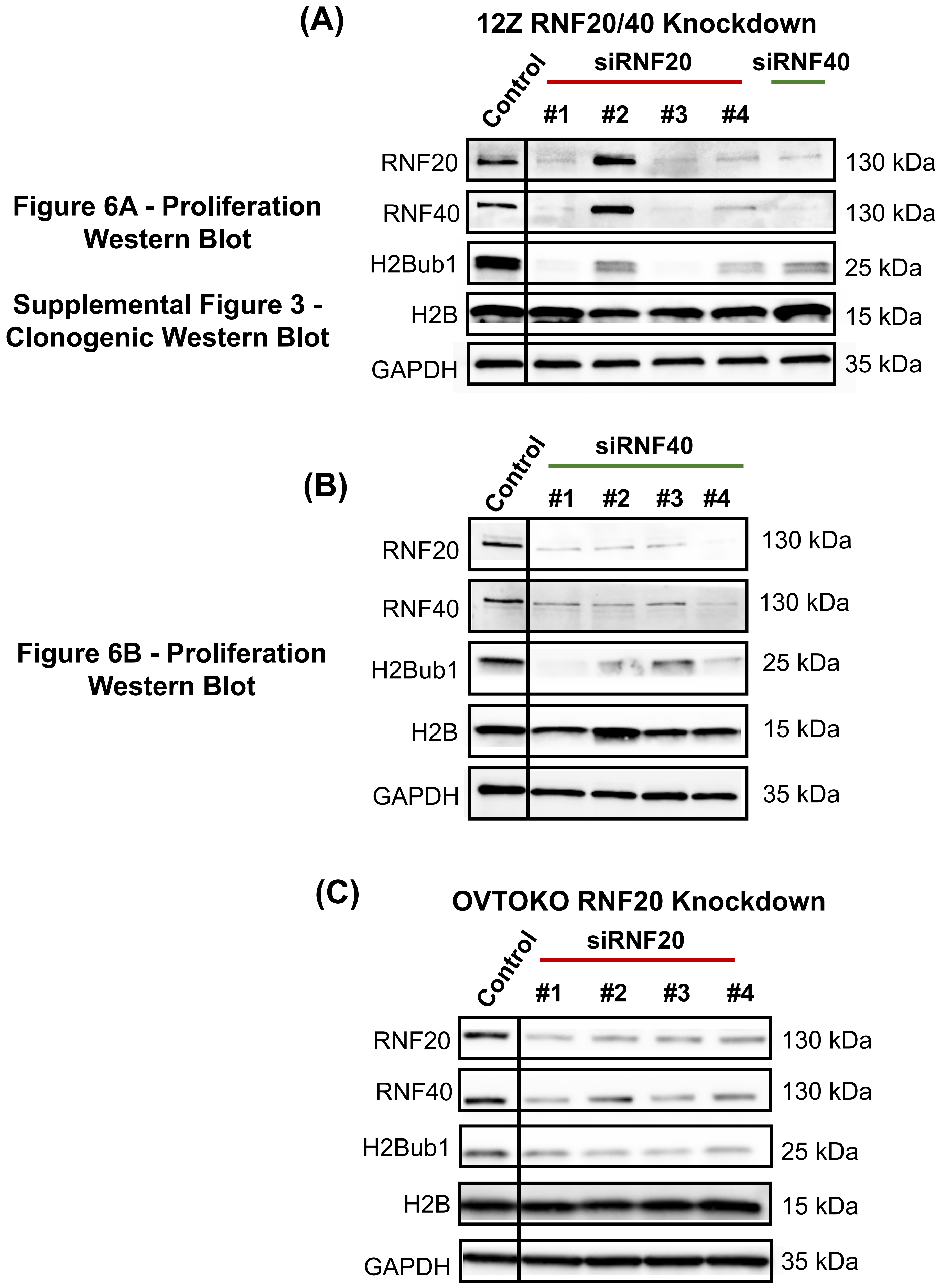

Supplemental Figure 8. Supportive WB. (A) RNF20/40 knockdown of proliferation assay (fig. 6A) and additional clonogenic analysis (supplemental fig. 3). (B) RNF40 knockdown of proliferation assay (fig. 6B). (C) Delineated siRNF20 pool. H2B and GAPDH serve as loading controls for all blots.

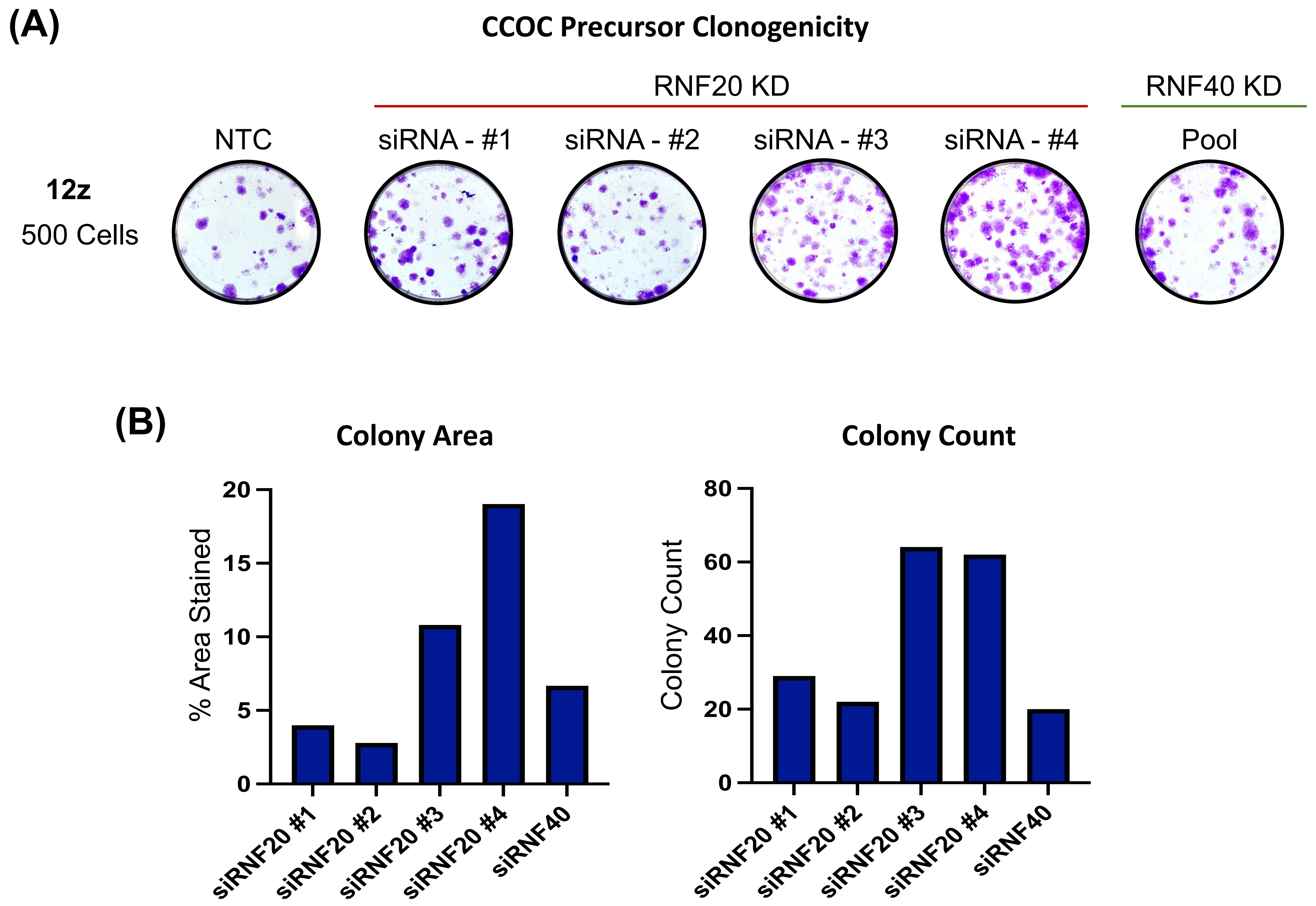

Supplemental Figure 9. Additional clonogenic analysis. (A) Clonogenic assay showcasing entire wells. (B) Quantification, colony area and count, of single experiment shown in panel A.

Categories	0	1+	2+	3+	Total N
Definition	composite score:<=1	composite score: >1, <=3	composite score: >3, <=5	composite score: >5	
RNF40	12	19	9	0	40
H2Bub1	28	11	1	0	40
H2B	0	14	18	8	40
UBR7	0	3	11	26	40

Supplemental Table 1. CCOC Composite breakdown (case count).

**Categories**	**0**	**1+**	**2+**	**3+**
Definition	composite score:<=1	composite score: >1, <=3	composite score: >3, <=5	composite score: >5
RNF40	30%	48%	23%	0%
H2Bub1	70%	28%	3%	0%
H2B	0%	35%	45%	20%
UBR7	0%	8%	28%	65%

Supplemental Table 2. CCOC Composite breakdown (percent of total).

Name	Method	Vendor	Cat #	Dilution	Clone	Antigen retrieval
H2Bub1	IHC	Cell Signaling Technology	5546	1:800	Monoclonal	
H2Bub1	WB	Cell Signaling Technology	5546	1:1000	Monoclonal	Anti-rabbit IgG, HRP-linked
H2B	IHC	Cell Signaling Technology	12364	1:2000	Monoclonal	
H2B	WB	Cell Signaling Technology	12364	1:1000	Monoclonal	Anti-rabbit IgG, HRP-linked
RNF40	IHC	Cell Signaling Technology	12187	1:200	Monoclonal	
RNF40	WB	Cell Signaling Technology	12187	1:500	Monoclonal	Anti-rabbit IgG, HRP-linked
UBR7	IHC	Bethyl Laboratories	A304-130A	1:250	Polyclonal	
UBR7	WB	Bethyl Laboratories	A304-130A	1:500	Polyclonal	Anti-rabbit IgG, HRP-linked
ARID1A	IHC	Sigma	HPA002123	1:250	Polyclonal	
RNF20	WB	Bethyl Laboratories	A300-714A	1:500	Polyclonal	Anti-rabbit IgG, HRP-linked
GAPDH	WB	Cell Signaling Technology	2118	1:1000	Monoclonal	Anti-rabbit IgG, HRP-linked

Supplemental Table 3. Antibody catalog.

Identifier	Target	Company	Catalog #
20 - #1	RNF20	Dharmacon	J-007027-05
20 - #2	RNF20	Dharmacon	J-007027-06
20 - #3	RNF20	Dharmacon	J-007027-07
20 - #4	RNF20	Dharmacon	J-007027-08
RNF20	RNF20	Dharmacon	L-007027-00-0010
40 - #1	RNF40	Dharmacon	J-006913-05
40 - #2	RNF40	Dharmacon	J-006913-06
40 - #3	RNF40	Dharmacon	J-006913-07
40 - #4	RNF40	Dharmacon	J-006913-08
RNF40	RNF40	Dharmacon	L-006913-00-0010

Supplemental Table 4. siRNA catalog.
